# Dual-Energy Computed Tomography for the Detection of Bone Edema-Like Lesions in the Equine Foot: Standing Horses and Cadaveric Specimens

**DOI:** 10.3390/vetsci12070614

**Published:** 2025-06-24

**Authors:** Jolien Germonpré, Ina Lorenz, Louis M. J. Vandekerckhove, Luc Duchateau, Torsten Diekhoff, Katrien Vanderperren

**Affiliations:** 1 Department of Morphology, Imaging, Orthopedics, Rehabilitation, and Nutrition, Faculty of Veterinary Medicine, Ghent University, 9820 Merelbeke, Belgium; 2 Pferdeklinik Bargteheide, 22941 Bargteheide, Germany; 3 Department of Radiology, Brandenburg Medical School, 15562 Rüdersdorf, Germany; 4 Department of Radiology, Immanuel Klinik Rüdersdorf, Seebad 82/83, 15562 Rüdersdorf, Germany

**Keywords:** bone marrow edema, bone marrow lesion, bone bruise, distal phalanx, coffin bone, navicular bone, distal limb

## Abstract

Dual-energy computed tomography (DECT) is a promising advancement for detecting bone edema-like lesions (BME). However, its application in horses remains limited. The aim of this study was to evaluate DECT virtual-non-calcium (VNCa) imaging in the equine foot and establish which confounding factors could influence its applicability in clinical practice. The DECT VNCa map of 14 standing and 5 cadaveric (recumbent) cases with foot-related lameness was scored in consensus by two readers in comparison to MRI. Overall, 17/19 cases demonstrated BME on MRI, whereas 2 did not. Agreement between DECT VNCa and MRI was found in 15/19 feet (78.9%). Disagreement in 4/19 cases with BME was due to sclerosis (1/19), mild BME extent on MRI (2/19), or scan artifacts (1/19). The extent of BME was significantly underestimated using DECT VNCa compared to MRI (*p* = 0.016). No significant correlation was found between sclerosis score and the BME extent underestimation on DECT (*p* = 0.056). Between standing and post-mortem cases, there was no significant difference in the agreement between DECT and MRI (*p* = 0.53) or DECT VNCa image quality (*p* = 0.22). In conclusion, DECT VNCa effectively identified moderate and severe BME, and its use was feasible in standing positioning. In case of sclerosis, a case-by-case assessment is recommended.

## Simple Summary

Dual-energy computed tomography (DECT) is an emerging imaging technique used to detect bone marrow edema-like lesions (BME), but its use in horses remains limited. This study evaluated the use of DECT to detect BME in horses with foot lameness and investigated factors that could influence its application in clinical practice. DECT scans from 14 standing horses and 5 cadaveric feet were reviewed by two readers in comparison to magnetic resonance imaging (MRI), the current gold standard for BME detection. MRI showed BME in 17/19 cases. Agreement between DECT VNCa and MRI was found in 15/19 feet (78.9%). In the 4 remaining cases, DECT did not match MRI findings due to increased bone density (1/19), a mild BME extent (2/19), and image artifact (1/19). Overall, the extent of BME was significantly underestimated using DECT compared to MRI (*p* = 0.016). There was no significant relationship between increased bone density and BME extent underestimation on DECT (*p* = 0.056). Between the live and post-mortem DECT scans, there were no significant differences in image quality or agreement with MRI. In summary, DECT effectively detected moderate and severe BME, and its use was feasible in standing positioning. DECT images should be interpreted with caution in case of increased bone density.

## 1. Introduction

Recent developments in veterinary cross-sectional imaging have significantly advanced equine diagnostic imaging. This is particularly evident in the increasing use of standing computed tomography (CT) and magnetic resonance imaging (MRI) in horses [1,2,3,4].

Conventional or standard CT is the preferred imaging modality for assessing bone abnormalities in the equine distal limb, but its ability to detect bone marrow edema-like lesions (BME) is limited due to intra- and interindividual variations in trabecular bone density. As a result, MRI is often required as a complementary technique to accurately identify these lesions [5,6]. However, low-field MRI, which is most commonly used in clinical equine practice, remains a time-consuming process, regardless of whether it is performed under general anesthesia or in a standing position [4].

Dual-energy computed tomography (DECT) offers a novel approach that can help overcome this limitation of standard CT. During standard CT, a single attenuation value is acquired per voxel using one X-ray energy spectrum, but this value depends on multiple factors, including the effective atomic number, the mass density of the material, and incident photon energy [7,8]. Consequently, its ability to identify specific materials is limited. In contrast, DECT acquires two attenuation measurements per voxel at two different photon energy spectra. The ratio of the two attenuation measurements enables the specific characterization of the material composition using post-processing algorithms [9,10,11]. One clinically valuable post-processing technique is the virtual-non-calcium (VNCa) map, in which mineralized bone is subtracted to enhance the detection of underlying bone marrow changes, including BME.

The DECT VNCa map has been extensively investigated in human diagnostic imaging and validated for the detection of BME in multiple anatomical regions, including the spine, knee, and foot. Compared to MRI, DECT VNCa mapping demonstrates excellent diagnostic performance in humans, with a reported sensitivity of 81–94%, a specificity of 91–98%, and an overall accuracy of 90–91% [12,13,14,15,16,17]. However, its application in equine diagnostic imaging remains relatively limited, with only a few recent studies exploring its clinical potential [18,19,20].

Regarding the VNCa map, one recent proof-of-concept study demonstrated its feasibility for detecting BME in a case series of cadaveric equine feet [19]. Despite these promising initial results, several confounding factors remain unexplored that may limit the diagnostic performance of DECT VNCa mapping in horses.

Bone sclerosis is a known diagnostic pitfall in human medicine, as it can locally alter CT attenuation values, reducing diagnostic accuracy by making it difficult to distinguish sclerosis from BME [21,22]. This challenge may be even greater in the equine appendicular skeleton, which is physiologically denser. It may also have an impact on the detectability of mild or focal BME, as DECT is considered less accurate in the detection of mild BME [23]. Lastly, the influence of equine CT positioning, particularly standing versus recumbent scanning, has not been investigated: this may introduce motion artifacts that degrade image quality and diagnostic reliability. Collectively, these gaps underscore the need for further research to evaluate the clinical applicability and limitations of DECT VNCa mapping for BME detection in equine patients.

The aim of this study was twofold: (1) to assess the clinical utility of DECT VNCa imaging for detecting equine foot BME of differing etiopathologies, extensiveness, and grades of bone sclerosis, and (2) to compare its performance in standing horses versus recumbent, post-mortem specimens. We hypothesized that the detectability of BME on DECT VNCa imaging was reduced in cases of sclerosis or mild BME, and could vary with the type of BME. Additionally, scan artifacts related to differing recumbency and motion were expected to negatively impact the diagnostic quality of the DECT VNCa maps in standing cases.

## 2. Materials and Methods

### 2.1. Study Design

In this prospective comparative imaging study, seventeen standing cases and five post-mortem cadavers were collected. For live horses, informed consent for the use of patient data was provided by the owner. The use of live animals in this study was not subjected to prior ethical approval by law (EU directive 2010/63/EU), as the diagnostic imaging data was obtained “below threshold”, i.e., as part of the diagnostic examination of the patient. For cadaveric specimens, ethical approval was waived by the ethical committee, based on European legislation (EU directive 2010/63/EU), as the tissues were obtained post-mortem. All horses presented with foot-related lameness or pathologies. Cases were collected at two institutions: four cadaveric specimens were collected at Ghent University (Belgium), and all standing cases plus one cadaveric specimen were collected at Pferdeklinik Bargteheide (Germany). Standing cases were included if scheduled for standing MRI and CT of the foot as part of their diagnostic work-up for foot-related lameness or pathologies during the same inpatient stay. Cadaveric specimens were obtained post-euthanasia due to a poor clinical prognosis, with euthanasia performed for reasons unrelated to this study.

For each case, the following details were recorded: status (live or post-mortem), age, sex, breed, limb location, anamnesis, and lameness duration and intensity. Lameness duration until diagnostic imaging was categorized as acute (onset up to 2 weeks ago), subacute (onset 2 weeks to 1 month ago), or chronic (onset more than 1 month ago). Lameness intensity was graded based on anamnesis or clinical examination as mild, moderate, or severe/walking lame. For cadaveric specimens, time between death and imaging was noted. Follow-up information was collected for standing horses when available. BME was categorized for each case as traumatic, infectious, or reactive/inflammatory based on the combination of anamnesis, radiographic findings, CT, and MRI findings.

### 2.2. Diagnostic Imaging

#### 2.2.1. Magnetic Resonance Imaging (MRI)

A standard protocol for the equine foot region was performed using the 0.25 T Vet-MR Grande (Esaote, Genoa, Italy) at Ghent University (Belgium) and the 0.27 T Hallmarq Standing Equine MRI at Pferdeklinik Bargteheide (Germany). T1-, gradient-echo T2- or T2-, and short-tau inversion recovery (STIR)-weighted sequences were acquired in at least one plane (see Table 1 for sequence details).

#### 2.2.2. Computed Tomography (CT)

Single-source DECT of the foot region was performed with a 320-row Canon Aquilion ONE Vision Edition at Ghent University (Belgium) and the 160-row Canon Aquilion Exceed LB at Pferdeklinik Bargteheide (Germany). For live horses, the foot region was scanned in a standing position. For post-mortem cases, the foot was scanned in lateral recumbency, corresponding to the positioning of a horse under general anesthesia. The same DECT protocol was used at both institutions: two volume scans were performed of the foot region in wide-volume mode, consisting of a high-energy (135 kVp) and a low-energy (80 kVp) scan. The tube current was set at 100 and 570 mA for the 135 and 80 kVp scan, respectively. The total scan length was achieved through the sequential acquisition of four consecutive 4 cm DECT volume scans. For a rotation time of 1 s, the total scan time for the Canon Aquilion ONE Vision Edition was 18.8 s (couch drive max. 16 cm/s), and was 14.4 s (couch drive max. 20 cm/s) for the Canon Aquilion Exceed LB. In a subset of cases, an additional standard helical CT scan was also performed with a similar scan protocol for both institutions (Ghent University: 135 kV, 300 mA, rotation time 0.75 s; Pferdeklinik Bargteheide: 135 kV, 310 mA, rotation time 0.60 s).

#### 2.2.3. Image Reconstruction

The DE soft tissue kernel (FC13) and standard bone kernel (from the DECT, and if available from helical CT) reconstructions, with a slice thickness of 0.5 mm, were calculated using iterative reconstruction (AIDR 3D standard). For each case, the DECT VNCa map was generated from the DE soft tissue reconstruction on the CT console at Ghent University (Belgium). The DECT VNCa maps were created using three-material decomposition post-processing software (‘DE Raw Data Analysis’, v. 8.9, Canon Medical Systems, Otawara, Japan), with a dual-energy gradient of 0.69 for calcium [24]. The object formula was 0/0 for water and −136/−106 for fat (80/135 kV).

#### 2.2.4. Image Analysis

Qualitative image analysis of all cases was performed in consensus by two readers, a European ECVDI diplomate (KV) and a doctoral candidate (JG) with three years of experience in DECT. The DE bone reconstruction (and the standard helical bone kernel CT when available) and the DECT VNCa map were reviewed independently and blinded to the MR images. All were reviewed in OsiriX (v. 12.5.2, Geneva, Switzerland) and retrieved from PACS (scoring overview see Table 2).

For each case, DE bone kernel reconstruction was initially assessed to determine the severity of bone sclerosis, beam-hardening, and remaining motion artifact using a 4-point Likert scale (0 = none, 1 = mild, 2 = moderate, and 3 = severe). The motion artifact was also only scored distal from the distal third of the proximal phalanx.

The DECT VNCa map was evaluated in grayscale with optional color mapping and in parallel to the standard bone CT blinded to MRI; free reformatting of slice thickness and window settings was allowed according to reader preference. BME on the DECT VNCa map was defined as a hyperdense lesion compared to normal bone marrow attenuation. BME was evaluated by location (distal phalanx, middle phalanx, proximal phalanx, and/or navicular bone) and graded for extent (none, mild, moderate, or severe). The highest extent score was recorded in the case of multifocal BME across multiple locations. The image quality of the DECT VNCa map was evaluated using a 4-point scale: 0 = non-diagnostic, 1 = poor, 2 = good, and 3 = excellent. Image quality was evaluated based on the degree of image noise, artifact streaks, and stitching artifact.

Next, MRI studies were evaluated as a reference for BME location and extent using the same grading scheme as for DECT VNCa. BME was defined as an increased fluid signal, evaluated as a region of STIR hyperintensity and corresponding T1-hypointensity [25,26].

Lastly, the anatomical location agreement between DECT VNCa and MRI in the location of BME was classified as either (1) agreement or (2) no agreement, both at the case level and at the individual bone level. Cases where BME was absent on both DECT and MRI were also classified as agreement. Cases of partial agreement, where BME was multifocal across multiple anatomical locations and not fully concordant between modalities, were classified as no agreement.

### 2.3. Statistical Analysis

BME location agreement between MRI and DECT was tested by the paired Wilcoxon rank sum test, i.e., using the difference between the MRI and DECT score (‘difscore’). The difscore, the MRI score, the DECT score, sclerosis, beam hardening, and image quality were compared between the two agreement groups (BME location agreement or no agreement) by the Wilcoxon rank sum test.

The relationship between difscore and sclerosis was assessed by Kendall’s correlation coefficient. The relationship of image quality with beam hardening and BME location agreement was assessed by Kendall’s correlation coefficient. The relationship between motion and BME location agreement was assessed by the Fischer exact test, and the two motion groups were compared with respect to image quality score by the Wilcoxon rank sum test.

The relationship between group (standing or cadaveric) and BME location agreement was assessed by the Fischer exact test. The MRI score was compared between the two walking lameness groups by the Wilcoxon rank sum test. The relationship between the MRI score and lameness intensity was assessed by Kendall’s correlation coefficient.

## 3. Results

### 3.1. Study Cohort Characteristics

Seventeen standing cases and five post-mortem cases were collected. Overall, 3 standing cases were excluded due to excessive motion during the CT examination, resulting in 19 cases being included from 18 horses (14 standing, 5 post-mortem cases). An overview of the details of the included cases is provided in the Supplementary Material (Table S1). All cases were from different horses, except for two standing cases, which represented an initial examination and a re-examination of the same horse. Of the 13 standing horses and 5 cadaveric specimens that were included, 10 were male and 8 were female, with a mean age of 9.8 ± 6.0 years old (all adult, minimum age of 4 years old). Fourteen horses were warmbloods and four were coldbloods.

At the case level, the following onset of lameness was reported: 9/19 presented with acute lameness, 4/19 presented with subacute lameness, and 6/19 presented with chronic lameness (ranging from 1 month to 6 years). Most cases had high-grade lameness, with lameness intensity categorized as follows: 11/19 were walking lame, 8/19 were moderately lame, and 0/19 mildly lame. The 14 standing cases included 13 front feet and 1 hind foot. The five cadaveric specimens included three front feet and two hind feet, with a mean post-mortem interval of 1.0 ± 1.2 days.

### 3.2. Image Analysis

**Figure 1:**
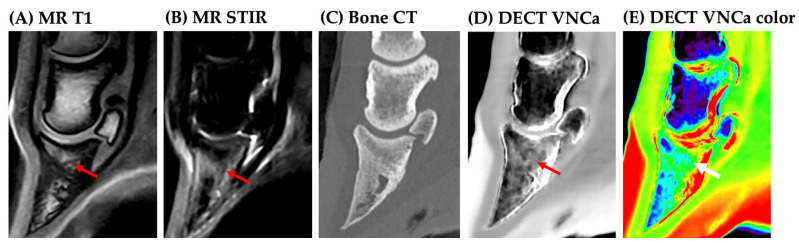
A 4-year-old Irish Cob with septic osteitis and a diffuse bone edema-like lesion (BME) of the distal phalanx (arrow) (post-mortem). (**A**) Diffuse T1-hypointensity of the distal phalanx; (**B**) diffuse short-tau recovery sequence hyperintensity of the distal phalanx; (**C**) standard bone CT—mildly increased bone density of the distal half of the distal phalanx; (**D**) DECT virtual-non-calcium map (VNCa) map—diffuse hyperdensity of the distal phalanx; (**E**) color-coded DECT VNCa map—BME is displayed in green, distinguishing it from normal fatty bone marrow, which appears blue.

**Figure 2:**
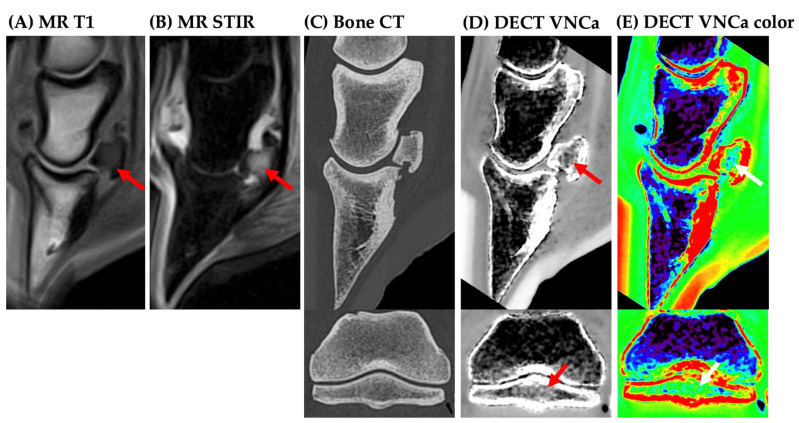
A **standing** 24-year-old Bradenburger with acute lameness of unknown origin and a severe diffuse bone edema-like lesion (BME) of the navicular bone (arrow). (**A**) Diffuse T1-hypointensity of the navicular bone; (**B**) diffuse short-tau inversion recovery sequence hyperintensity of the navicular bone; (**C**) standard bone CT (sagittal and transverse plane) within normal limits; (**D**) DECT virtual-non-calcium map (VNCa) map (sagittal and transverse plane)—diffuse hyperdensity of the navicular bone; (**E**) color DECT VNCa map (sagittal and transverse plane)—BME is displayed in green in contrast to the normal fatty bone marrow in the distal and middle phalanx (blue).

An overview of the DECT VNCa and MRI scoring is provided in the Supplementary Material (Table S2). On MRI, BME was observed in 17/19 cases, including 10 with severe, 4 with moderate, and 3 with mild BME extent. On MRI, BME was observed in the distal phalanx (7/17, Figure 1), navicular bone (3/17, Figure 2), or concurrently in both locations (7/17). No cases were identified with BME in the middle or proximal phalanx on MRI.

**Figure 3:**
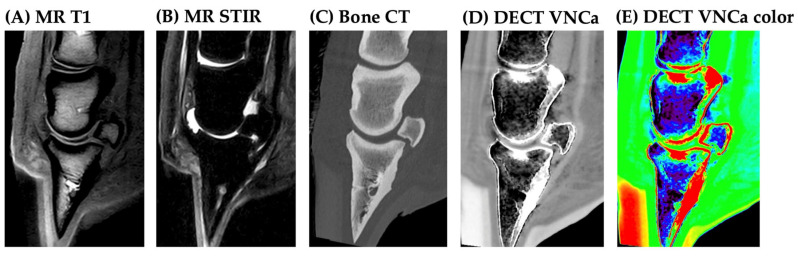
Equine foot without bone edema-like lesions (BME). (**A**) T1-weighted sequence; (**B**) short-tau inversion recovery sequence; (**C**) standard bone CT. (**D**) DECT virtual-non-calcium map (VNCa) map; (**E**) color-coded DECT VNCa map. No bone sclerosis on standard bone CT. On the DECT VNCa map, no BME/hyperattenuating regions are observed (green on color map) (apart from the hyperattenuating, physiological high-bone-density regions and cortices—green to red on color map). Yellow bone marrow appears blue on the color map.

At case level, both cases without BME on MRI (2/19) were true negatives on DECT VNCa (Figure 3). Overall diagnostic agreement between DECT VNCa and MRI was observed in 15/19 horses (78.9%), including cases with and without BME.

**Figure 4:**
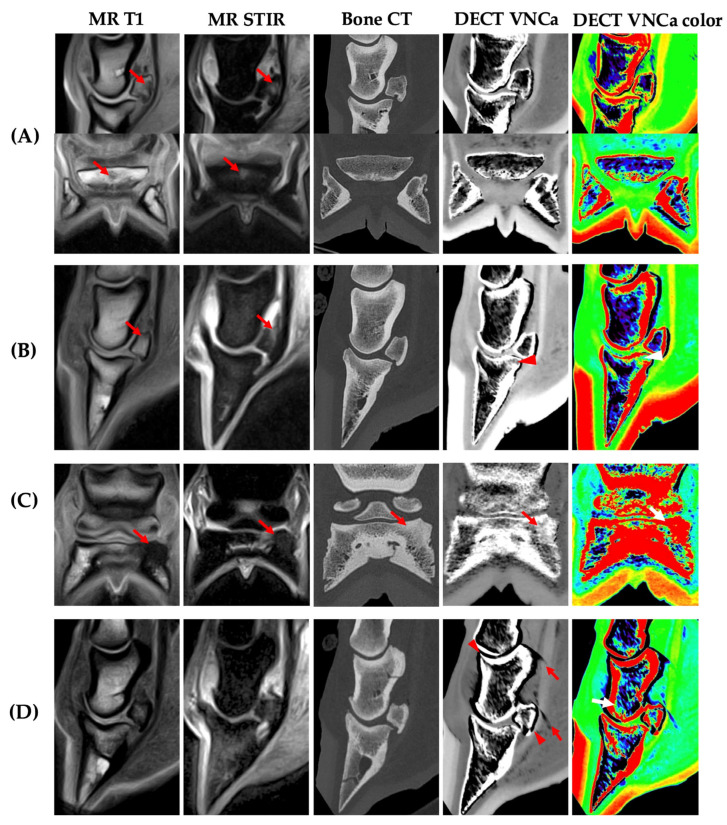
All four cases (**A**–**D**) without diagnostic agreement between DECT virtual-non-calcium (VNCa) imaging and MRI for bone edema-like lesions (BME). (**A**) Mild, focal, and well-delineated BME on MRI in the mid-sagittal aspect of the spongiosa and flexor cortex of the navicular bone (arrow) (sagittal and dorsal view). No sclerosis visible on standard bone CT. On the DECT VNCa map, no BME was observed. (**B**) Mild BME on MRI in the proximopalmar aspect of the spongiosa and flexor cortex of the navicular bone (arrow), without sclerosis on the standard bone CT. On the DECT VNCa map, readers note a false-positive BME observation at the attachment site of the impar ligament (arrowhead). (**C**) Moderate BME on MRI in the medial palmar process, with sclerosis of the same region on standard bone kernel CT (arrow). This sclerosis masked BME in this area on the DECT VNCa map. (**D**) Severe, diffuse BME in the distal phalanx and navicular bone on MRI. No sclerosis was visible on the standard bone kernel CT. On the DECT VNCa map, BME was observed; however, readers note a false-positive BME observation in the dorsodistal aspect of the middle phalanx (white arrow). Note the beam-hardening streaks (red arrow), increased noise, and volume-stitching artifact (in line with arrowheads) on the DECT VNCa map.

At the individual bone level, the results of the contingency table analysis for the detection of BME for each individual bone are presented in Table 3 (distal phalanx), Table 4 (navicular bone), and Table 5 (middle phalanx). At the patient level, false-positive and false-negative observations for BME were found in 4/19 cases (all standing), which are all presented in Figure 4. This was attributable to several confounding factors. The first was the mild BME extent: although there was no significant difference found in BME extent on MRI between the agreement and disagreement group (*p* = 0.62), two of three cases with a mild BME extent on MRI were missed on DECT (Figure 4A,B). However, the BME extent score at the patient level was significantly underestimated on DECT VNCa imaging compared to MRI (*p* = 0.016); among the cases with BME on MRI (17/19), 58.9% were assigned a lower BME extent score on DECT VNCa. No cases were assigned a higher BME extent score on DECT VNCa imaging compared to MRI.

**Figure 5:**
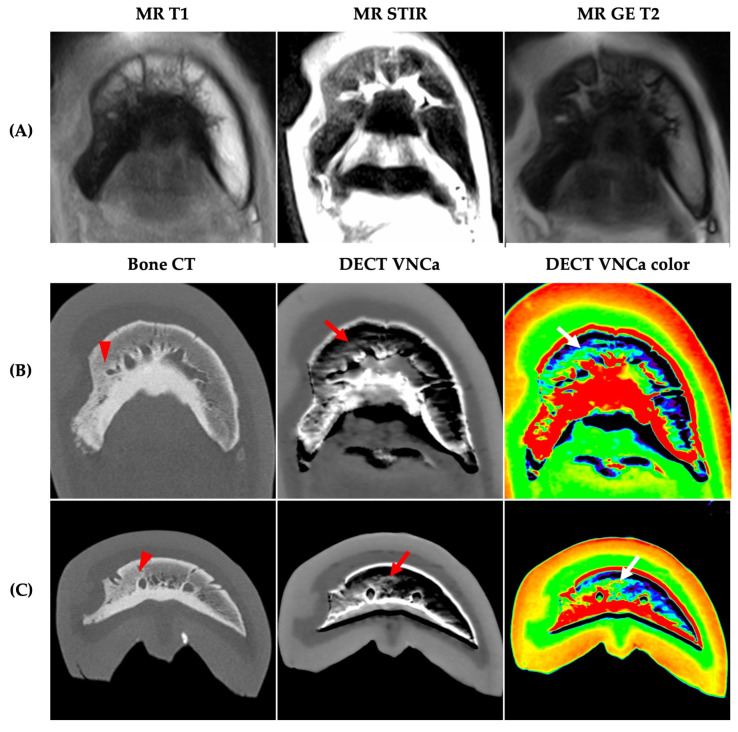
Standing horse, 9 years old, male, left front foot distal phalanx, infected keratoma with history of hoof wall infection. Bone edema-like lesion (BME) could be observed on the DECT virtual-non-calcium (VNCa) map despite the presence of sclerosis. (**A**) MRI (T1 and GE T2 hypointense with STIR sequence hyperintensity) showed BME of the medial palmar process, extending towards the mid-sagittal plane (**B**,**C**) Transverse planes with bone sclerosis in the medial palmar process on standard bone CT (arrowhead). On the DECT VNCa map, the BME extends further towards the mid-sagittal plane (hyperattenuating or green on the color-coded map) (arrow).

Bone sclerosis was observed in the majority of the cases (14/19) (73.7%), with a mean bone sclerosis score of 1.3/3 ± 1.0 on a 0–3 scale (score 0: 5 feet; score 1: 6 feet; score 2: 6 feet; score 3: 2 feet). Readers subjectively identified this as the main confounding factor in the evaluation of DECT VNCa maps. However, the majority of cases were still correctly identified (Figure 5) and sclerosis score did not differ significantly between the agreement (1.33/3 ± 1.03) and disagreement group (1.00 ± 1.15) (*p* = 0.63). Diagnostic agreement between DECT VNCa imaging and MRI due to bone sclerosis occurred in only one case involving the distal phalanx (Figure 4C).

Although no overall correlation was found between the sclerosis score and the difference in the BME extent score between DECT VNCa and MRI (r = 0.41, *p* = 0.056), the BME extent was underestimated on DECT VNCa in 5 out of 8 cases with a sclerosis score of 2 or 3. It is also important to note that the two cases with severe sclerosis (score 3) also had marked BME on MRI. All cases with moderate sclerosis (score 2) had an MRI BME extent score of moderate or severe, apart from one case with no BME detected on MRI.

Apart from bone sclerosis, physiologically high bone density was also a confounding factor. In one case, high bone density at the attachment site of the impar ligament on the distal phalanx resulted in a false-positive BME observation (Figure 4B).

Another false-positive observation of BME was found in the dorsodistal aspect of the middle phalanx (Figure 4D). This standing case had a low image quality score for the VNCa map (score 1/3) and a moderate beam-hardening artifact (2/3 score). Additionally, increased image noise and volume-stitching artifact were present.

Overall, beam-hardening artifact differed significantly between the agreement (0.13/3 ± 0.35) and disagreement group (1.00/3 ± 0.58) (*p* = 0.01). Among all 19 cases, 4 standing cases received a beam-hardening artifact score greater than 0 (score 1: 4 feet; score 2: 1 foot; score 3: none). Moreover, 3 out of the 4 cases without agreement between DECT VNCa and MRI had a beam-hardening artifact score ≥ 1/3. Although there was a significant difference in beam-hardening score, no significant difference in image quality score was found between the agreement and disagreement groups (*p* = 0.33). There was also no correlation between the DECT VNCa image quality and the beam hardening score (r = −0.334, *p* = 0.14).

The mean image quality of the DECT VNCa map across all cases was 2.3/3 ± 0.6. There was no significant difference in image quality between standing cases (2.2/3 ± 0.6, *n* = 14) and post-mortem specimens (2.6/3 ± 0.6, *n* = 5). No significant relationship was found between image quality score and motion (*p* = 0.53); a minor motion artifact was observed on the DE bone reconstruction in only three standing cases (all scored 1/3), yet all were assigned an image quality score of 2/3.

### 3.3. Standing Versus Recumbent Group

No significant differences were found between the standing and cadaveric specimens for MRI BME extent score (*p* = 0.36), beam-hardening artifact (*p* = 0.72), and image quality (*p* = 0.22). Between standing and cadaveric horses, there was no statistically significant differences in diagnostic agreement for BME location between DECT VNCa and MRI (*p* = 0.53).

### 3.4. Effect of BME Type

No significant relationship was found between BME extent and either lameness intensity or lameness duration. Cases were categorized based on BME type, including 7 infectious cases, 7 traumatic cases, 2 reactive/inflammatory cases, and 3 cases of unclear BME origin. Four out of 7 infectious cases were penetrating nail injuries.

Two of the four cases without diagnostic agreement, both characterized by mild BME not associated with sclerosis or scan protocol artifacts, were classified as infectious BME (Figure 4A,B). In both instances, focal lysis of the navicular bone with prominent vascular channels was observed. The remaining two cases without diagnostic agreement were classified as traumatic BME (the case affected by marked sclerosis; Figure 4C) and as infectious BME (the case affected by scan protocol artifact; Figure 4D).

### 3.5. Follow-Up

In addition to the cadaveric specimens that were euthanized due to poor clinical prognosis, 7/14 standing cases were euthanized after the completion of the study due to poor clinical prognosis. Follow-up information was unavailable for two cases. Two of the remaining five horses are still in rehabilitation. The other three horses that successfully returned to work exhibited moderate to no BME. The standing horses exhibiting either marked BME or walking lameness were euthanized, except for one case in each subgroup that is still in rehabilitation. Lastly, for the two cases without BME, the standing case returned to work and has been to shows again; the other was a cadaveric specimen that had already been euthanized due to a tendon lesion.

## 4. Discussion

The aim of this study was to evaluate the use of DECT VNCa imaging in the equine foot compared to the gold standard—MRI—for detecting BME under variable conditions and confounding factors, reflecting cases typically encountered in clinical practice.

BME was observed on MRI in 17/19 horses. Overall, there was a good diagnostic agreement between DECT VNCa imaging and MRI for identifying the location of the BME; agreement was observed in 15/19 horses (78.9%). Although this was a small cohort, the level of agreement is consistent with reports from human studies. Various confounding factors inherent to equine anatomy, pathophysiology, and scan protocols were identified across the cohort. Diagnostic disagreement between DECT VNCa and MRI in detecting BME location in 4/19 horses was attributable to both false-positive and false-negative findings.

Firstly, the overall BME extent score was significantly lower on DECT VNCa imaging than on MRI: 58.9% of cases with BME were assigned a lower BME extent score on DECT VNCa imaging. This likely explains why diagnostic agreement between MRI and DECT VNCa imaging was not reached in two out of three cases with a mild BME extent on MRI; both cases showed a focal, concomitant low T1-hypointensity and STIR hyperintesity. Although the BME extent score on MRI did not significantly differ between the agreement and disagreement groups (*p* = 0.62), this non-significant difference may have resulted from the fact that disagreement in the BME location on DECT VNCa and MRI was not primarily due to mild BME alone. This observation aligns with the human literature, reporting a possible decrease in the diagnostic accuracy of DECT VNCa imaging in cases with subtle BME on MRI [23,27,28,29]. The findings of this study suggest the existence of a potential direct relationship between BME extent on DECT VNCa imaging and the degree of hypointensity in the MR T1-weighted sequence. This emphasizes the importance of diagnosing BME based on the characteristic combination of STIR-hyperintensity and corresponding T1-hypointensity [25,26]. T1-hypointensity is essential, as increased STIR signal alone is commonly observed in the equine foot in both lame and sound horses, although it is typically more extensive in lame horses [30]. Additionally, a potential explanation for why mild BME was not detected on DECT VNCa in certain cases could be its localization within the navicular bone, where the limited volume of cancellous bone may reduce detectability. Furthermore, the anatomical localization of the BME (adjacent to the flexor cortex) might have been an obscuring factor, as BME should ideally be evaluated at least 2 mm away from the bone cortex [31].

Secondly, physiologically high bone density resulted in one false-negative observation in the distal phalanx at the attachment site of the impar ligament. Previous work by this research team already described this as a possible confounding factor in the interpretation of DECT VNCa imaging in the equine foot [19]. Awareness of these physiological variations is essential for accurate DECT assessment, particularly in regions where mechanical stress may physiologically increase bone density.

Thirdly, increased bone density or bone sclerosis was observed in the majority of cases on standard bone CT (73.7%), which exhibited mild sclerosis or greater. This prevalence of sclerosis was in line with expectations as both acute and chronic cases were included in this study, and an association has been reported between the presence of BME and concomitant increased bone formation in this region in horses [32].

Although most sclerotic cases were correctly identified, sclerosis was identified as the main limiting factor when evaluating the DECT VNCa map. This aligns with the human literature describing the confounding effect of sclerosis on the visualization and interpretation of BME in DECT VNCa imaging; Diekhoff et al. (2019, 2022) and Foti et al. (2020) emphasized that increased bone density may mimic or mask underlying BME due to their similar hyperdense appearance, thereby reducing diagnostic accuracy [23,28,33]. An inverse relationship between bone density and BME detectability has been proposed, with BME more readily visualized in areas exhibiting less sclerosis [34]. Consequently, it is recommended that DECT VNCa maps should interpreted with caution and in parallel with standard CT reconstructions to minimize the risk of misdiagnosis.

Although the exclusion of cases with severe sclerosis has been suggested to improve diagnostic accuracy, Deppe et al. (2023) reported that diagnostic accuracy did not increase when such cases were excluded [21]. This observation is consistent with the findings in this study; sclerosis score did not differ significantly between the agreement and disagreement group. BME could still be identified when extended beyond the sclerotic region or in the case of mild sclerosis with remaining trabecular space; in contrast, in areas lacking intertrabecular space, the absence of residual fat likely limits the contrast necessary for BME detection on DECT VNCa maps.

Despite the promising results achieved in the presence of sclerosis, it is important to note that cases with moderate to severe sclerosis were generally associated with a moderate to severe BME extent on MRI in this study; this may have facilitated the detectability of BME in this subgroup.

No overall correlation was found between the sclerosis score and the difference in BME extent scores between DECT VNCa and MRI. However, BME extent was underestimated on DECT VNCa in 3 out of 8 cases with a moderate sclerosis score or higher; this may indicate a non-significant trend (*p* = 0.056) toward a potential correlation. A larger sample size may be required to confirm this hypothesis. We hypothesize that the detectability of BME in equine feet with sclerosis may vary depending on the study population and the extent of BME, and the exclusion of cases with severe sclerosis may still be justified in selected contexts.

Lastly, beam hardening was also a confounding factor for image quality, leading to a lower image quality score for the VNCa map due to increased noise and artifact streaks at the level of the joints. Overall, beam hardening was significantly increased in the disagreement group. This suggests that the presence of beam-hardening artifacts is likely an important factor in obscuring or mimicking BME on the DECT VNCa map. All four cases with disagreement were standing cases. It is hypothesized that this is due to the more oblique positioning of the foot in standing CT compared to recumbent imaging, where the foot is usually positioned straight and in the CT gantry isocenter. When the foot is scanned obliquely, several factors may contribute to reduced image quality: off-center positioning in the CT gantry, the potential lack of automated dose modulation, the X-ray beam needing to travel through a longer section of dense bone, and oblique transitions through joint spaces. Although no significant effect of beam-hardening was observed on DECT VNCa image quality, this may still be a trend to investigate further as the non-significant results could be explained by the low number of cases with beam-hardening artifact (5 standing: 4 mild and 1 moderate).

It was hypothesized that sequential, single-source volume DECT acquisitions would need to be entirely free of motion to be of diagnostic value. Although the software on the CT console corrects for micromovement, the high- and low-kVp datasets need to be exactly paired. Yet, only 3/14 standing cases were scored with mild presence of motion artifact and all were still assigned a good image quality score. Also, no significant relationship was found between image quality score and motion. This may be a result of the DECT scan protocol used in this study: four sequential dual-energy volume scans were acquired, which were then combined through volume stitching to produce the full DECT volume. A key disadvantage of this approach is the relatively long total acquisition time, which can be challenging for standing horses. However, it offers the advantage that motion during scanning only affects the specific DE volume being acquired at the time, along with minor stitching artifacts between adjacent volumes. This is clinically beneficial, as motion artifacts in standing CT were typically more pronounced in the proximal regions of the distal limb (e.g., the fetlock), which are physically less supported during scanning. This limits motion artifacts to the first proximal DECT volume scan, unlike a single 16 cm DECT volume scan (e.g., Canon Aquilion ONE Vision Edition), where any motion would compromise the entire volume, though it has a much shorter acquisition time (2.4 s).

For each case, the BME was categorized as traumatic, infectious, or reactive/inflammatory in origin. The two cases for which no diagnostic disagreement was found due to the characteristics of the BME lesion (i.e., excluding confounding factors such as sclerosis or scan protocol artifacts) were both classified as infectious BME. This may be attributed to the fact that osteomyelitis exhibits a more subtle increase in HU on DECT VNCa imaging than other types of BME [35]. However, given that a large proportion of cases in the cohort were classified as infectious BME (7 out of 19), and considering the small sample size, this may be coincidental. As such, no significant associations or trends between BME type and DECT VNCa detectability could be established within the scope of this study. This observation then aligns with human reports; although most DECT VNCa diagnostic accuracy studies focus on traumatic BME, the modality has also demonstrated the reliable detection of non-traumatic BME [36,37,38,39].

Normal bone marrow is a dynamic entity: the bone marrow of younger patients needs to be interpreted with caution, as immature red bone marrow has a higher bound water content and may mimic pathological BME on DECT VNCa imaging [13,28,39]. Although red marrow in the equine foot is presumed negligible due to the faster conversion rate in peripheral appendicular bones, only adult horses were included in this study for this reason [40,41]. Also, to the authors’ knowledge, bone marrow reconversion in the equine foot is undocumented and was considered negligible, since the foot is likely the last affected, being the most peripheral joint [42].

The DECT VNCa map was evaluated in grayscale with optional color mapping, allowing the free adjustment of slice thickness and window settings according to reader preference. The readers preferred a thicker slice (minimum 3 mm in sagittal plane) to decrease image noise on the DECT VNCa map. Note that DECT VNCa figures were made to match the imaging plane of MRI. The DECT VNCa map allows for multiplanar reformation with freely adjustable slice thickness.

Certain limitations were acknowledged in this study. Although the number of horses included was sufficient to address the primary hypotheses, the relatively small sample size limited the statistical power for subgroup analyses, such as those based on BME type or varying grades of sclerosis. This limitation raises the risk of Type II errors, where potential differences or associations may go undetected. Therefore, a larger cohort would facilitate more subgroup analyses and may validate trends observed. Future studies could stratify findings across a spectrum of sclerosis severity, similar to the approach taken by Wang et al. (2013) in assessing diagnostic accuracy in human vertebral bodies with less than 50% sclerosis [34]. Power calculations should be conducted in future studies to determine adequate sample sizes for such stratified analyses, ensuring sufficient sensitivity to detect clinically meaningful differences.

Only qualitative analysis of BME was performed for this study. The qualitative analysis method was chosen because, when evaluating BME on DECT VNCa imaging across multiple anatomic regions with different physiological high-density values, several different cut-off values would be required to perform quantitative analysis of DECT numbers [23]. Additionally, the presence of different grades of bone sclerosis, which were observed to both imitate and overlap the BME region, is also expected to alter the DECT numbers. Due to these confounding factors, the number of positive findings would not have been sufficient for each anatomical region per sclerosis grade to generate a reliable cut-off value for BME in the equine foot. The BME cut-off value is also likely to be dependent on the DECT technology used and post-processing parameters [22,43]. Apart from these reasons, qualitative assessment (85% sensitivity, 97% specificity) has been reported to be more accurate than quantitative assessment (84% sensitivity, 88% specificity) [44].

The aim of the study was to identify potential confounding factors in the detection of BME through DECT VNCa imaging. As it was not designed as a diagnostic accuracy study, the study cohort lacked negative cases at the case level. Nevertheless, the same research group has previously demonstrated the application of DECT VNCa in normal equine feet, and the BME-negative cases in this study were also correctly interpreted as normal on DECT [19]. The predominance of front feet in the dataset, with only one standing hind foot included, was coincidental due to case availability. The image quality of the VNCa maps was evaluated alongside agreement with MRI findings, though future studies could explore diagnostic preference between grayscale and color-coded maps. In this study, both were assessed together, but readers noted a subjective increase in confidence when evaluating the color-coded map. Both readers had several years of DECT experience, and as shown by Deppe et al. (2023), diagnostic performance tends to improve with reader expertise [21]; future studies may benefit from a multi-reader approach including varied experience levels and disciplines. Finally, while this study focused on the equine foot, BME is also prevalent in the equine fetlock joint [32]. Follow-up studies could apply this methodology to the fetlock region, where similar confounding factors, such as naturally higher bone density, motion, and beam-hardening artifacts or scan positioning may influence DECT VNCa interpretation to a different extent.

## 5. Conclusions

This study demonstrated that DECT VNCa imaging can effectively identify BME in the equine foot, particularly when BME was moderate to severe in extent. Agreement between DECT VNCa and MRI was found in 15 out of 19 cases (78.9%). In the remaining cases, discrepancies were attributed to the presence of bone sclerosis (1/19), mild BME extent on MRI (2/19), and motion artifact (1/19). While the BME extent was significantly underestimated on DECT VNCa compared to MRI (*p* = 0.016), no significant correlation was found between sclerosis grade and underestimation (*p* = 0.056).

No significant trends were observed between the BME type and DECT detectability within the scope of this study, and further research with larger case numbers is warranted. DECT was feasible in standing horses in comparison to recumbent cadaveric specimens, with no significant differences in image quality (*p* = 0.22) or agreement with MRI (*p* = 0.53). These results support the application of DECT in a standing, clinical setting, although motion artifacts remain an important consideration.

In cases with marked bone sclerosis, interpretation should be conducted cautiously, as areas with complete loss of intertrabecular space may limit BME detection. Reader experience may play an important role in minimizing diagnostic uncertainty, especially in the presence of confounding factors such as sclerosis and motion artifacts.

## Figures and Tables

**Table 1:** Overview magnetic resonance imaging (MRI) sequence details at both institutions: Esaote Vet-MR Grande (0.25 T) and the Hallmarq Standing MRI (0.27 T). STIR: short tau inversion recovery-weighted; GET2: gradient-echo T2-weighted; TR: repetition time (ms); TE: echo time (ms); TI: inversion time (ms); slice: slice thickness in mm.

	Esaote Vet-MR Grande				Hallmarq Standing MRI			
Sequence	TR	TE	TI	Slice	TR	TE	TI	Slice
T1	22	9	-	0.43	24	7	-	3
STIR	4480–5580	30	70–75	3.5	1500	8	90	5
T2	4260	100	-	4	1848	81	-	5
GET2	1925	22	-	3.5	34	13	-	3

**Table 2:** Overview of scoring categories for dual-energy computed tomography (DECT) virtual-non-calcium (VNCa) map and MRI in detection of bone edema-like lesions (BME).

Category	Modality	Scores	
Bone sclerosis	DE Bone CT	(0) none, (1) mild, (2) moderate, (3) severe	
Beam-hardening artifact	DE Bone CT	(0) none, (1) mild, (2) moderate, (3) severe	
Motion artifact	DE Bone CT	(0) none, (1) mild, (2) moderate, (3) severe	
Image quality	DECT VNCa	(0) non-diagnostic, (1) poor, (2) good, (3) excellent	
BME location	DECT VNCa	distal phalanx navicular bone	middle phalanx proximal bone
	MRI		
BME extent	DECT VNCa	(0) none, (1) mild, (2) moderate, (3) severe	
	MRI		
BME location agreement	DECT VNCa MRI	Yes or no	

**Table 3:** Contingency table for bone edema-like lesions (BME) in the distal phalanx.

BME	MRI(+)	MRI(−)
DECT VNCa(+)	13	1
DECT VNCa(−)	1	4

**Table 4:** Contingency table for bone edema-like lesions (BME) in the navicular bone.

BME	MRI(+)	MRI(−)
DECT VNCa(+)	8	0
DECT VNCa(−)	2	9

**Table 5:** Contingency table for bone edema-like lesions (BME) in the middle phalanx.

BME	MRI(+)	MRI(−)
DECT VNCa(+)	0	1
DECT VNCa(−)	0	18

## Data Availability

The original contributions presented in this study are included in the article/Supplementary Materials. Further inquiries can be directed to the corresponding author.

## Supplementary Materials

The following supporting information can be downloaded at: https://www.mdpi.com/article/10.3390/vetsci12070614/s1, Table S1: Overview of case characteristics; Table S2: Overview of the qualitative scoring.

## Abbreviations

The following abbreviations are used in this manuscript:

AIDR	Adaptive Iterative Dose Reduction
BME	Bone edema-like lesion
CT	Computed tomography
DECT	Dual-energy computed tomography
ET	Echo time
FC	Filter convolution
GE	Gradient echo
IT	Inversion time
kVp	Kilovoltage peak
MRI	Magnetic resonance imaging
PACS	Picture archiving and communication system
PM	Post-mortem
RT	Repetition time
STIR	Short-tau inversion recovery
VNCa	Virtual-non-calcium
