# Supplementary material for: Dual-Energy Computed Tomography for the Detection of Bone Edema-Like Lesions in the Equine Foot: Standing Horses and Cadaveric Specimens
Source: Vet Sci. 2025 Jun 24;12(7):614. doi: 10.3390/vetsci12070614 (PMC12298723; doi:10.3390/vetsci12070614)
Supplement: Supplementary file 1 [file vetsci-12-00614-s001.zip › vetsci-3703710-supplementary.pdf]

Table S1. Overview of case characteristics, including state (live or post-mortem), age (in years), sex (male/female), breed, foot, and lameness duration and severity. Legend: PM: post-mortem; M: male; Fe: female; R: right; L: left; F: front; H: hind. BME: bone marrow oedema-like lesion; RX: radiographic examination; MRI: magnetic resonance imaging. CT: computed tomography.

Foot	State	Age	Sex	Breed	Foot	Lameness duration	Lameness severity	Description	Diagnostic imaging before inclusion	Follow-up
1	live	6	M	Hannoveraner	R-F	acute	walking lame	Medial palmar process fracture	RX one month prior: within normal limits. MRI one month prior: BME in medial palmar process without fracture. Remained lame until re-examination study.	Re-examination at 3.5 months following stall rest: no visible lameness. RX: good fracture healing. Controlled exercise (four weeks of walking) was initiated, followed by gradual return to training.
2	pm	11	M	Islandic horse	R-F	chronic	walking lame	Distal tendon sheath effusion with pain during flexing	RX: within normal limits. No pulse, no reaction to the hoof tester, very painful to palpation on the sesamoid bones.	euthanized
3	live	24	Fe	Bradenburger	R-F	acute	walking lame	Lameness of unknown origin	RX within normal limits	euthanized
4	live	6	M	Holsteiner	L-F	acute	moderate	Proximal resorption/lysis of the navicular bone	RX: prominent vascular channels at the distal navicular border	The horse underwent 4 months of rest and medical treatment. Two follow-up MRIs confirmed resolution of BME. Control CT remained unremarkable aside from multiple vascular channels at the distal border. The horse had returned to work.
5	live	7	M	Hannoveraner	R-F	acute	moderate	Traumatic injury with displacement of shoe	RX within normal limits	MRI 4 months later: resolution of BME. The horse was sound and had returned to work.
6	live	9	M	Hannoveraner	L-F	acute	moderate	infected keratoma with history of hoof wall infection	RX: previous infected keratoma surgery	went to rehabilitation, no follow up information.
7	live	9	Fe	Quarter Horse	R-F	chronic	moderate	Navicular disease	RX within normal limits	euthanized (due to tendon lesions)
8	live	9	Fe	Standardbred	R-F	acute	walking lame	navicular bone fragmentation with focal lysis	Radiographs within normal limits	euthanized
9	live	8	M	Mecklenburger	L-F	acute	moderate	Penetrating nail injury	RX: nail penetration channel visible. MRI/CT: CT showed severe progressive osteolysis	euthanized
10	live	4	M	Westfalen	L-F	chronic	walking lame	Unknown trauma; osteitis of the distal phalanx	RX within normal limits	See foot 11

11(*)	live	4	M	Westfalen	L-F	chronic	walking lame	Re-examination: Unknown trauma; osteitis of the distal phalanx	Re-examination of Foot 10: CT/MRI: progression of the lesions	euthanized
12	pm	7	Fe	Belgian warmblood	L-H	subacute	walking lame	Hoof abscess with laminitis and distal phalanx sinking	RX: suspicion of abscess at the level of the toe and lateral quarter	euthanized
13	pm	5	Fe	Belgian Draught	R-H	acute	walking lame	Penetrating nail injury; chronic palmar process fracture	RX: Penetration tract with metallic probe at the level of the distal aspect navicular bone	euthanized
14	pm	4	M	Irish Cob	L-F	chronic	walking lame	Septic osteitis of the distal phalanx	RX: chronic laminitis, large solar abscess, associated osteitis margo solearis distal phalanx	euthanized
15	pm	24	Fe	Fjord	R-F	subacute	walking lame	Penetrating nail injury	RX: penetration channel from the caudal third of the sole to the navicular bone. Suspicion of osteomyelitis of the navicular bone, involvement of DDFT, DIP joint and navicular bursa.	euthanized
16	live	8	M	Holsteiner	R-F	acute	moderate	Recurrent fracture medial palmar process	RX: coffin bone fracture	still in rehab
17	live	15	Fe	Holsteiner	L-F	chronic	moderate	Osteitis of the distal phalanx	Not available	no follow up information
18	live	11	M	Zangersheider	R-F	subacute	moderate	Intra-articular fracture palmar process	RX: referral, intra-articular fracture palmar process	back in training and has been to shows again
19	live	4	Fe	Hannoveraner	R-H	subacute	walking lame	Penetrating nail injury	RX: penetration channel was visible, but no bone or tendon lesion	euthanized

(*) re-examination of foot 10 (same horse).

Table S2. Overview of the qualitative scoring.

Case_ID	group	Motion	Image quality	Beam Hardening	Sclerosis	BME_MRI	BME_DECT	BME location MRI	Agreement	BME type
1	live	0	3	0	2	moderate	none	distal phalanx	no	Traumatic
2	pm	0	3	0	0	severe	severe	distal phalanx, navicular bone	yes	Reactive/ inflammatory
3	live	0	3	0	0	severe	severe	navicular bone	yes	Unclear
4	live	1	2	1	0	mild	none	navicular bone	no	Infectious
5	live	0	2	0	1	moderate	moderate	distal phalanx	yes	Traumatic
6	live	0	2	0	2	moderate	moderate	distal phalanx	yes	Infectious
7	live	1	2	1	1	severe	moderate	distal phalanx, navicular bone	yes	Unclear
8	live	0	2	1	0	mild	mild	navicular bone	no	Infectious
9	live	0	1	2	2	severe	moderate	distal phalanx, navicular bone	no	Infectious
10	live	1	2	0	2	moderate	moderate	distal phalanx, navicular bone	yes	Traumatic
11	live	0	3	0	3	severe	moderate	distal phalanx, navicular bone	yes	Traumatic
12	pm	0	2	0	3	severe	moderate	distal phalanx	yes	Traumatic
13	pm	0	3	0	1	none	none	absent	yes	Reactive/ inflammatory
14	pm	0	3	1	1	severe	severe	distal phalanx	yes	Infectious
15	pm	0	2	0	0	severe	severe	distal phalanx, navicular bone	yes	Infectious
16	live	0	2	0	2	severe	moderate	distal phalanx, navicular bone	yes	Traumatic
17	live	0	2	0	1	mild	mild	distal phalanx	yes	Unclear
18	live	0	2	0	2	none	none	absent	yes	Traumatic
19	live	0	3	0	1	severe	severe	distal phalanx	yes	Infectious
